# Towards Improving the Prenatal Diagnosis of Congenital Heart Disease: This Time, for Mother’s Health∗

**DOI:** 10.1016/j.jacadv.2022.100127

**Published:** 2022-10-28

**Authors:** Lindsay Freud, Nimrah Abbasi

**Affiliations:** a Division of Cardiology, Department of Paediatrics, The Hospital for Sick Children, University of Toronto, Toronto, Ontario, Canada; b Division of Maternal-Fetal Medicine, Department of Obstetrics and Gynaecology, Mount Sinai Hospital & Ontario Fetal Centre, University of Toronto, Toronto, Ontario, Canada

**Keywords:** fetal echocardiography, peripartum outcome, severe maternal morbidity, ultrasound screening

**Figure:**
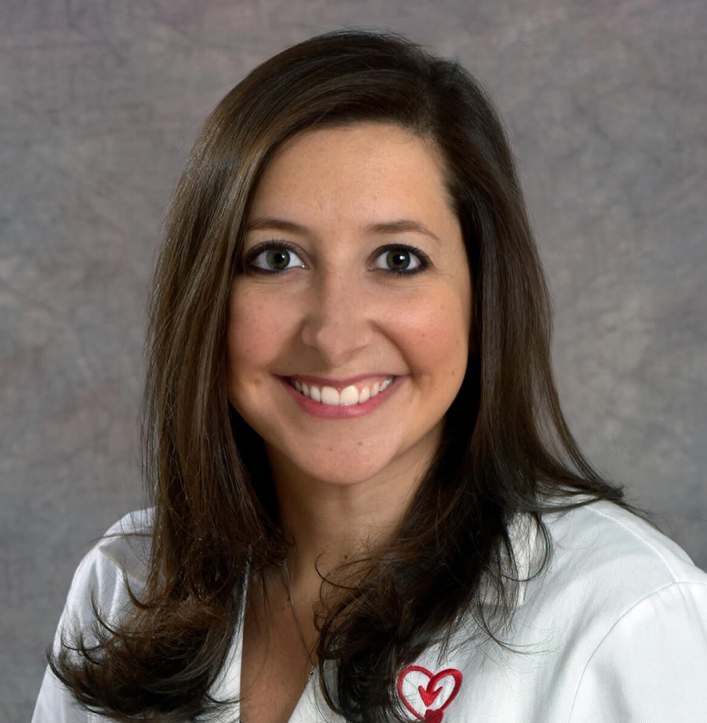

While a great deal of attention has been paid to neonatal outcomes of pregnancies complicated by fetal congenital heart disease (CHD), maternal outcomes have remained relatively unexplored. In this issue of *JACC: Advances*, Tseng et al^1^ report a novel independent association between fetal CHD and severe maternal morbidity (SMM) using state-wide administrative data from Ohio. Their findings have highly relevant clinical implications for specialized delivery planning and, in our opinion, highlight the potential importance of a prenatal diagnosis of fetal CHD for optimizing the mother’s health. The study also raises salient questions regarding the biologic mechanisms that underlie both fetal CHD and maternal morbidity.

In this study linking birth certificate and birth defect surveillance data in Ohio, 682,929 mothers had live-births between 2011 and 2015. Consistent with prior epidemiologic series,^2^ 0.85% had isolated fetal CHD, defined as CHD without a known genetic syndrome or extracardiac anomaly. It is unknown whether the CHD was diagnosed prenatally or postnatally. Persistent fetal shunts, such as an atrial septal defect or patent ductus arteriosus, were appropriately excluded. SMM was defined as maternal intensive care unit admission during delivery hospitalization, uterine rupture, unplanned operative procedure after delivery, or need for blood transfusion. Due to limitations of the dataset, the nature of the unplanned operative procedure, ie, simple dilation and curettage vs life-saving hysterectomy, was not available, and many of the known major causes of SMM per the Centers for Disease Control, including cardiovascular disease, hemorrhage, infection, venous thromboembolism, and hypertensive disorders of pregnancy, were not evaluated.

Although SMM may have been underestimated throughout the cohort, the rate of SMM was higher in pregnancies complicated by fetal CHD than that in pregnancies with no fetal anomalies (3.6% vs 1.9%, *P* < 0.001). Since the risk factors for fetal CHD and adverse maternal outcomes overlap, the authors adjusted for maternal age, race/ethnicity, body mass index, diabetes, hypertension, and smoking status. Fetal CHD remained independently associated with SMM (adjusted relative risk: 1.81 [95% CI: 1.58-2.08]). Using sensitivity analyses with cesarean section (CS) and premature delivery as mediators, the risk was attenuated but persisted. In addition, fetal CHD was associated with increased maternal transfer prior to delivery.

There are several important limitations to acknowledge. Pregnancies complicated by fetal CHD were more likely to have both limited (1-5 visits) and late prenatal care (>20 weeks), 10.5% vs 6.9% and 30.0% vs 25.7%, respectively (both *P* < 0.001). Although substandard prenatal care would be expected to impact both the prenatal diagnosis of CHD and the peripartum outcome, this variable was unfortunately not retained in the final multivariable model of SMM. In addition, since the time of diagnosis of CHD was unknown, the potential—and highly probable—protective effect of a prenatal diagnosis could not be ascertained.

Nevertheless, the authors’ findings allow us to consider the benefit of a prenatal diagnosis of fetal CHD for the mother’s health. A prenatal diagnosis of fetal CHD offers multiple advantages, including the opportunity for education and counseling, comprehensive diagnostic evaluation, decision-making, possible fetal therapy, and specialized delivery planning. The literature to date focuses on delivery planning to reduce neonatal morbidity and mortality. Such delivery planning often involves maternal fetal medicine specialists, which also likely mitigates risks to the mother.

For example, a higher rate of CS (40.8% vs 29.7%, *P* < 0.001), including primary CS (25.2% vs 17.3%, *P* < 0.001), was noted among pregnancies complicated by fetal CHD in this study. The indication for CS was not provided, which may have been for maternal/obstetric reasons or due to fetal intolerance of labor, but it also may have been to facilitate delivery timing or for perceived safety of CS over vaginal delivery by providers and/or patients. CS may not have been necessary in these latter scenarios and may be associated with many of the major known causes of SMM that were not accounted for in this study. Moreover, there is currently no evidence to support CS for the fetus/neonate with CHD. Coordination of care at an experienced perinatal center would offer the opportunity to discuss both timing and mode of delivery, ultimately optimizing both maternal and neonatal outcomes.

Despite both the theoretical and proven benefits of prenatal diagnosis of CHD, it remains woefully underdiagnosed. Only approximately 50% of CHD is diagnosed prenatally.^3^ While single ventricle defects are more commonly detected due to an abnormal screening 4-chamber view, transposition of the great arteries (TGA), another critical form of CHD, is only prenatally diagnosed 50% to 60% of the time. This is largely because prenatal diagnosis of TGA requires evaluation of the outflow tracts, which was only recently included in the international second-trimester ultrasound screening guidelines in 2013.^4^ The screening 3-vessel view (3VV), a transverse view of the fetal mediastinum that demonstrates the size and spatial arrangement of the superior vena cava, aorta, and pulmonary artery, was just incorporated into the international screening guidelines this year,^5^ and many national societies, including the American Institute of Ultrasound in Medicine, continue to recommend this view only if technically feasible.^6^ The 3VV, in our opinion, is both technically easier to obtain and cognitively easier to interpret than the outflow tract views. Widespread education and adaptation of the 3VV will hopefully pave the way toward increasing prenatal diagnosis rates of CHD in the coming years.

It is no coincidence that prenatal diagnosis rates of fetal CHD, particularly TGA, are related to distance from a tertiary care center; that is, further distance has been associated with lower likelihood of a prenatal diagnosis in North America.^7^ This reflects not only socioeconomic status but also poor access to high-quality prenatal care and ultrasound screening. Distance from a tertiary care center was not included as a variable in this study. Maternal zip code, if available, would allow for calculation of distance, as well as a more comprehensive index of social deprivation, and should be considered in future studies. Not surprisingly, the authors found higher rates of maternal transfer among pregnancies complicated by fetal CHD, presumably to a higher level of care. Although the indication for referral—maternal or fetal—was unknown, urgent transfer of care can be anxiety-provoking for patients and costly for health care systems. It underscores the importance of providing equitable access to high-quality prenatal care to identify and moderate perinatal risk factors, including fetal CHD.

Simple forms of fetal CHD tend to have even lower prenatal diagnosis rates than complex CHD since they may not be readily detected on ultrasound screening. Nevertheless, the authors found an intriguing observation: there was no difference in SMM between fetuses with simple and complex CHD. Furthermore, fetuses with a particularly simple form of fetal CHD, ventricular septal defect, had higher rates of SMM than fetuses with no anomalies. These findings suggest a shared pathogenesis between fetal CHD and SMM that may not be entirely alleviated by enhanced prenatal care and a timely diagnosis of fetal CHD. The direction of the effect is unclear, and the mechanism is, as of yet, not understood. It may include genetic and epigenetic components and likely hinges upon the central role of the placenta—a unique organ whose developmental origins and function derive from a complex interplay between maternal and fetal factors. Placentas of pregnancies complicated by fetal CHD are known to have more vascular malperfusion lesions than controls, as well as evidence of chronic inflammation.^8^ Translational research evaluating angiogenic growth factors, such as placental growth factor, and placental pathology in pregnancies complicated by fetal CHD and SMM may provide insights into this important association.

In conclusion, the work by Tseng et al^1^ enables us to ponder yet another advantage of prenatal diagnosis of fetal CHD—the opportunity to focus on the mother’s health and mitigate SMM. Equitable access to high-quality prenatal care, incorporation of comprehensive fetal cardiac views on screening ultrasound, and delivery planning at a tertiary (or quaternary) care center with maternal fetal medicine and CHD specialists are necessary cornerstones to improve both maternal and neonatal health in pregnancies complicated by fetal CHD.

## Funding support and author disclosures

The authors have reported that they have no relationships relevant to the contents of this paper to disclose.
